# Book Reviews

**Published:** 1813-07

**Authors:** 


### CRITICAL ANALYSIS

### *OF RECENT PUBLICATIONS*

IN THE

### DIFFERENT BRANCHES OF PHYSIC, SURGERY, AND MEDICAL PHILOSOPHY.

### *Edinburgh Medical and Surgical Journal, No.* XXXIII.

I. *Case of Congenital Cataract; with some Observations on the Means of Artificially Dilating the Pupil in the Operations of Extracting and Depressing the Cataract. By* John Henry Wishart, Surgeon.

THIS case of congenital cataract differs so little from many others which have lately been laid before the public, that it will be useless to repeat it: serving, however, as the motive for giving a short historical account of the employment of certain narcotic vegetables for dilating the pupil, it is entitled to our consideration.

The accidental acquirement of the fact of the *Atropa Belladonna*, when applied to or near the eye, causing a temporary dilatation of the pupil, interesting and valuable as it is, remained long without any useful practical appropriation. The account given by Ray, (*Hist. Plant. L.* xiii. *c.* 23, *p.* 680) and copied by Van Swieten into his Commentaries on the Aphorisms of Boerhaave, (*Aphor.* 1060) and even the observations of Galen, (*Method. Medend. lib.* iii. *cap.* 11, *tom.* x. *p.* 53. *Charter*) that opium, mandrake, and henbane, produce the same effect, excited no attention beyond the simple fact; nor was this property, diffused, probably, among a large family of plants, turned to any account, until a gentleman who was preparing the extract of the *Belladonna*

for for Reimarus at Hamburgh, chanced to apply the juice of the recent herb to his eye. The effect produced continued three weeks, and the circumstances of it are related by Reimarus.

"Quod attinet ad oculum tuum, olim, inter conscindendam *Atropæ Belladonnæ* herbam, ita affectum, ut per aliquod tempus fere visu privaretur, probe memini. Deprehendi nempe iridem ejus oculi adeo resolutam et patentem, ut fere dimidiæ tantum lineæ latitudo ambitus superesset, ampla vero choroideæ nigrities transpareret. Jam, cum effectum similem ab ore assumta *Belladonnæ* observari nossem, non dubitavi, quod ipse tu quoque suspicatus fueras, externe applicatum venenum idem efficere posse et ab insperso inter parandum succo paralysin illam ortam, quam tamen duraturam haud esse speravi. Interius jam acetum quoddam concentratum assumpseras, in cujus usu pergendum esse ratus sum: suasi tantum, ut simul ad irritandos nervos exterius volatilis oleosi spiritus vapores oculo ægro admitterentur. Sive igitur sponte, seu his adjuvantibus medicamentis, factum est, ut sensim et visus oculo et contractio pupillæ redirent. Ego vero mecum reputans, istam *Belladonnæ* vim haud spernendo in chirurgia usui esse posse cogito. Nempe cum in excidenda lente crystallina, sicut pelluciditatem amiserit, haud leve impedimentum objiciat irritatio et contractio nimia pupillæ, adeo ut sæpe iris per educendam lentem laceretur; quidni succo *Belladonnæ*, paralysin illi innocuum per aliquod tempus inducimus? nec solum extractio lentis inde longe facilior obtineretur, sed et simul illud incommodi, ne oculus repentino lucis sensu læderetur."

In a former number we noticed this discovery, and the claim Reimarus and Grasmeyer have for bringing it into notice. That other vegetable narcotics possessed this property was a reasonable inference *à priori*, and experience has shown this to be the fact. Mr. Wishart has collected the substance of what is known on the continent respecting the employment of the hyoscyamus as an application to the eye. Professor Himly, of Gottingen, (*Ophthalmalogische Beobachtungen und Untersuchungen. Bremen*, 1801) after the application of a solution of the extract of hyoscyamus, found the eye exactly in the same state as in a case of complete amaurosis: the iris was motionless, and so far drawn back that it formed a ring scarcely a line in breadth, with its inner edge turned a little backward, so that its anterior surface was concave toward the central point; the pigmentum nigrum, at the bottom of the eye, had not its usual black color, but was greyish. Oleum cajeputi rubbed on the eyebrow restored the contractile power of the iris. A dram of the extract of hyoscyamus, in an ounce of water, is the proportion employed by Prof. Himly: of this solution a few drops are put into the eye, and kept there a short time by bending the head backwards. It occasions no pain, and no perceptible redness. The paralysis of the pupil comes on in an hour, and continues five or six hours: its action is so local that the iris only is affected, the retina never suffering. It will be obvious, from this fact, that the effect produced on the eye by the hyoscyamus does not exactly resemble amaurosis; but is an operation, *sui generis*, on the iris alone, and that its influence does not extend to the optic nerve or its expansion at the bottom of the eye. From this property in the hyoscyamus, Professor Himly deduces the following conclusions:

1st, It affords a certain test whether the cataract adheres to the iris.

2dly, It assists in the diagnosis of the capsular, lenticular, fluid, and firm cataract. It will also assist in determining whether, in those cases of cataract in which the patient sees colored points and bodies, that circumstance arises from a defect in the retina, or from some peculiar state of the lens. If this state of vision arises from some peculiarity in the opake lens, then will the dilatation of the pupil diminish it; but if from a disease of the retina, by the transmission of a greater number of rays, this morbid symptom will be increased. This is a practical fact of considerable importance, as decisive, frequently, of the propriety of undertaking the operation for cataract.

3dly, In cases of common cataract it may prove a palliative, by inducing a state of the iris more favorable for the transmission of rays of light to the retina.

4thly, It procures vision in many cases of opacity of the cornea situated immediately before the pupil, by the simple dilatation of the iris beyond the verge of the opacity. It may be a question, however, if the constant or even frequent use of this narcotic as a palliative in this case and in section 3d, may be admissible, on the possibility of its producing permanent paralysis of the iris, and even of the retina.

5thly and 6thly, The professor points out the advantages to be derived from the hyoscyamus in the various operations for cataract; not merely as affording the means of ascertaining the condition and degree of morbid change, but as an auxiliary during the operation.

"In many cases the application of the hyoscyamus facilitates the operation for cataract; when, for instance, even after a sufficiently large incision, the cataract does not pass out, from the pupil being too much contracted, and remaining so, though the eye be left at perfect rest. In this case, however, he recommends the precaution of not operating during the greatest dilatation of the pupil, as there would be great danger of causing a prolapsus of the vitreous humor, as as it would receive too little support from the iris. He, therefore, allows the greatest effect to be over, and operates when the pupil has already contracted, and the iris acquired a slight degree of motion. The application of the hyoscyamus will also prove beneficial, if we operate according to Beer's method,* as the cataract, passing out along with its capsule, requires greater yielding of the pupil; and, if partial adhesions of the iris to the capsule are to be separated, the wider the pupil, the more safely can the necessary means be used for their separation; and likewise, if the capsule is opake, it can be more completely destroyed if the pupil be previously in a state of dilatation, and the danger of wounding the iris with the knife, in a case of a very flat cornea, would thereby probably be diminished.

"The dilatation of the pupil by the hyoscyamus, would be of great use in Conradi's† method of effecting the absorption of the cataract by opening the capsule; as, in this case, the success depends entirely on the free access given to the aqueous humor to the lens,—consequently, the capsule must not be opened merely in one small point, but a large

* "This mode is very accurately described in the Encyclopedia Britannica, article Surgery.

† "From the well known fact of the crystalline lens being often gradually absorbed, whether it be of a hard or soft consistence, if the capsule is opened, and the humors allowed to come into free contact with it, Conradi was led to propose the following simple operation: 'A small lancet-shaped cataract needle is introduced through the cornea, exactly as the knife in extraction, only a very little farther distant from the iris. The point is passed through the pupil, and when the capsule is sufficiently opened, the needle is withdrawn from the eye, which is bound up loosely for two or three days, as in general, after that time, so slight a wound of the cornea is quite imperceptible, and then we wait for the absorption of the cataract. The needle does not require to be quite so long as Richter's cataract knife, and it should not be more than a line and a half or two lines in breadth. Its thickness should be very small, merely to give the blade a sufficient degree of firmness; it must be sharp on both sides for nearly one half of its length.' Conradi adds, 'It is unnecessary to dwell on the evident advantages of this operation; it is much more easily performed than any other. From this very slight and trifling wound of the insensible cornea, no bad symptoms are to be dreaded, which partly occur during extraction and depression, partly follow after these operations. If, after eight or twelve weeks, the cataract is not absorbed, any other operation may be performed as easily as if this puncture had not been made. The patients have lost nothing but the time; and, as they have in general been many years blind, it is not of much consequence, a few weeks, more or less, in order to make the trial of freeing them from their disease by a safe and easy method.'—See Arnemann's Magazin für die Wundartzneiwissenschaft, 1 B. 1. St. Göttingen, 1797."

incision incision must be made in it, which can be readily done if the pupil be previously dilated."

7thly, The local application of the hyoscyamus is particularly useful in contraction of the pupil, which is not accompanied with an adhesion of the iris to the capsule.

To the excerpts made from this work of Professor Himly, which has been translated into French with the mistake of *hyoscyamus* being rendered *belladonna*, Mr. Wishart adds some facts from the late Professor Schmidt of Vienna, who used these two narcotics both externally and internally.

"In the month of May, 1803, Professor Schmidt operated on twenty-nine patients of both sexes, affected with cataract, in the general hospital at Vienna; on twenty eyes by extraction, and twenty-two by depression of the lens. Of these twenty-nine persons, twenty-six recovered their sight. Eight were selected as the subject of his experiments. In five he tried the application of the solution of the hyoscyamus four hours before the operation; in three he used the inspissated juice of the leaves of the belladonna as many hours before the operation. The phenomenon of the retraction of the iris (dilatation of the pupil) occurred in all the eight patients, but the degree of the dilatation was different in each individual. The difference of age and sex had no influence on it.

"Of these eight patients, he operated on three eyes with the couching-needle, through the sclerotic and choroid coats, and six by extraction through the cornea. Of the three that were couched, in a woman the iris expanded completely during the puncturing with the needle, and the pupil assumed its smallest dimensions. Of the six operated on by extraction, the same phenomenon was observed in one woman and one man; and in two others, the expansion of the iris evidently took place, but was not complete. To one of these three, where the complete expansion of the iris came on during the incision of the cornea, the belladonna was applied. Of these nine cases, only one was attacked with iritis after extraction, and consequent closure of the pupil.

"Of the twenty-two eyes operated on by extraction, there were only two where the cataract was followed by a slight effusion of the vitreous humor, and this only occurred in the eyes subjected to experiment. It ought also to be observed, that this protrusion of the vitreous humor occurred without any strong pressure having been applied to the eye-ball; that the cataract was purely lenticular, and there was no unusual adhesion of the capsule; and lastly, in both cases, the belladonna had been used. Professor Schmidt lastly remarks, that, in all the six patients, it appeared to him as if the cataract was more *unwilling* to come out, (if the expression may be allowed;) and that he was convinced, that neither the size of the incision, nor of the opening of the capsule, nor any sort of adhesion, could have the most distant share in producing this circumstance."*

* "See Ophthalmalogische Bibliothek von Himly und Schmidt, St. II. B. I. Jena, 1803."

It

It is of some practical importance to ascertain whether the narcotic principle in these plants differs in degree only, or in some quality or property by which it acts on the iris. Mr. Wishart says he has generally, of late, used the hyoscyamus in preference to the belladonna, because it excites less pain. In one case of cataract he used the hyoscyamus as a palliative for a year, without observing any bad effects to arise from this long and continued use. It will be worth while to ascertain if the belladonna possesses, in an equal degree with the hyoscyamus, the valuable property of drawing back the iris into the eye, so as to reverse its usual form, making that part concave which is naturally convex. We consider the action of the narcotic principle of vegetables upon vitality generally as of great interest, and in the particular cases now mentioned to be highly important. With this impression we may be pardoned for laying before our readers some facts in the preceding account possibly known to many of them; but an object of so much utility cannot too often come under observation.

II. *Dissection of an Albuminous Concretion which was found in the Cavity of the Thorax, loosely adhering to the Pleura Pulmonalis; with some Observations on the Diseases of the Serous and Synovial Membranes. By* JAMES WARDROP, Surgeon.

On opening the thorax of an adult a whitish tumor was observed, loosely adhering to the pleura. It very much resembled a piece of cartilage, in color, transparency, elasticity, and firmness. Its form was globular, but slightly flattened, its surface polished, and it was about the size of a Spanish hazel-nut. It adhered to the lung in a few points by white membranous bands. It cut precisely like common cartilage, and the section showed it to be composed of a number of concentric laminæ, surrounding an osseous central portion. The bony nucleus was small, of a dark brown, and composed of concentric layers.

This case gives Mr. Wardrop occasion to make some observations on what he denominates albuminous concretions, and on diseases of the serous and synovial membranes. He contends that the formation of cartilaginous or osseous substances in various cavities is not to be considered as the growth or elongation of cartilage or bone; or to be a deposit or decomposition of the synovial fluid, or of that fluid which moistens serous cavities, but that they are the sequela of previous inflammation; and he states his hypothesis in the following passage:

"It is very probable that the membrane lining those cavities in which the concretions are formed, has been at one period or another affected with inflammation. When a serous or synovial membrane becomes inflamed, in place of mere exhalation which is naturally going on, a distinct quantity of fluid is collected, from which an albuminous deposit is formed. In most instances this albuminous matter is re-absorbed; but when a portion remains, it acquires a very considerable degree of firmness, as we observe in adhesions formed by the pleura and peritonæum converted into bone. If a piece of albumen be in this manner left loosely attached to the membrane, and if a quantity of fluid be at the same time collected in the cavity, the albumen will be modelled into a particular shape, from the motion and pressure to which it is exposed. Whilst the albumen remains attached, it may thus undergo various changes in form, and any fresh attack of inflammation, however slight, may add to its bulk; but when it is once detached, by its slender peduncle giving way, no future change can take place, unless that of absorption; for, when loose and unconnected, it must be considered as separated from the system, and liable to be acted on by the absorbents as a foreign substance. It has been briefly noticed, that these concretions are either entirely composed of a soft substance, or of a substance partly soft and partly osseous; or they are altogether composed of bone. The same may be noticed of the common adhesions formed between serous and synovial membranes, and of the changes which those membranes themselves undergo from chronic inflammation. These too are sometimes merely ligamentous, but in other instances they are ossified; thus showing, that adhesions and concretions ought to be considered as the effect of the same morbid action, and merely varieties of disease, in place of distinct morbid alterations of structure."

III. *Case of Hernia Cerebri. By* Daniel Pring, Surgeon.

The points of this case are shortly these:—A man received on the 8th of April a blow on the head, by which a fracture of the cranium was occasioned. No symptoms of cerebral derangement ensued for some days; but the sore in the scalp was ill-conditioned, and by passing a probe into the wound, some spicula of bone were discovered. At this time, the seventh day after the accident, there was great prostration of strength, and the pulse was no more than 50, soft, and extremely weak. On the twelfth day there was great increase of irritation, and symptoms of pressure on the brain had appeared. On the thirteenth day there was a protrusion in the wound esteemed to be fungous, but which proved to be a portion of cerebral substance, constituting the *Hernia Cerebri.*

As there had been evidently a secretion of pus on the surface of the brain for some distance, within the cavity of the cranium, from the wound, this hernial state of the brain, by shutting up the exit for the pus, became the accidental cause cause of the pressure and irritation. By degrees the patient got well, and on the 3d of June the wound was quite cicatrized.

It is presumed by the author, that the spicula of bone, which had inflamed the membranes and subsequently produced sloughing and hernia, should have been early removed, though no symptoms of cerebral derangement were present. In particular instances this may be proper, and where it can be done without much exposure; but as a general principle of practice the propriety of it remains doubtful if not objectionable. It may be a question if exposure of the dura mater is the surest way to prevent its inflaming. The symptoms of pressure arising from the confinement and accumulation of pus within the cranium, may in these cases be removed by pressing down the hernial protrusion with a smooth spatula, so as to give exit to the fluid at sufficiently frequent intervals. Immediate mischief will thus be prevented; and, as far as our experience goes, we can say, that by gentle and judicious pressure the protruded part of the brain will be gradually confined within the cranium.

### IV. *On the good Effect of Ipecacuan and Laudanum in Dysentery. By* George Playfair, Surgeon.

Half a drachm to a drachm of ipecacuan with from 30 to 60 drops of laudanum were given, confining the patient for some hours to an horizontal posture. It usually happened, that after the medicine was taken, no inclination for stool was experienced for many hours, the patient being, during that time, free from pain; several loose motions then took place, but unmixed with blood, and without tenesmus. It sometimes happened that several loose motions succeeded the medicine in a very short time, and none afterwards; and that the bowels were even costive the day after the medicine had been taken.

Seldom more than one dose was required, but when any symptoms remained, a repetition next day was sufficient to cure the disease. This treatment was adopted at the commencement of the disease; afterward it was not beneficial, because the stomach became too irritable to retain the medicine. The following cases will explain, in a measure, the disease, and illustrate the treatment.

" W. Troy, a private of his Majesty's 65th regiment, was, on the evening of the 11th of April, attacked with dysentery. The symptoms were severe, and accompanied with fever. He had given him half a drachm of ipecacuan, with 35 drops of laudanum, and had several loose easy motions in the course of the night. On the morning of the 12th, the griping pain had almost left him; he felt easy in his bowels, and during the whole of the day had only one motion; but he complained of slight head-ache, his pulse was rather quick, and his skin hot. I gave him three grains of calomel, with a little rhubarb, and he had for his diet sago, with an allowance of wine. 14th, He had no griping pain, head-ache almost gone, felt a sensation of soreness all over the abdomen, and had no stool since the 12th. I gave him 15 grains of rhubarb and 30 grains of magnesia, which procured him three easy motions, after which he seemed well in every respect. His head feeling light and giddy, had a few doses of bark and port wine, and on the 16th was discharged well. The medicine in this case caused no nausea whatever."

"—— Dawson, 24th of April, complained of severe griping and looseness of the bowels, with constant inclination to go to stool, and much tenesmus; his evacuations consisted of mucus mixed with blood. I gave him one drachm of ipecacuan with 60 drops of laudanum. He vomited the first, but retained a repetition of the dose. Had only one stool in the night of the 24th, and the griping had entirely left him. He had for his food a strong decoction of barley. On the 26th he had two or three stools, which he described as consisting entirely of blood, but had none in the evening or during the night. The morning of the 27th complained only of flatulence; his face seemed slightly swelled, and had been so ever since he had the first dose of the medicine. Had complained of head-ache on the 26th, but which had then left him; 28th he had no complaint remaining."

V. VI. *Cases of Hydrophobia. By* Mr. TYMON, Dr. BERRY, *and* Dr. SHOOLBRED.

These cases have previously appeared in the preceding volume of our Journal.

VII. VIII. *On the Introduction of the Depletory Method of Cure in the Tropical Fever.*

In the ardent fever of the tropics, the interruption of inordinate vascular action by the combined powers of bleeding, purging, and the affusion of cold water, forms the general indication of cure. It is the object of the first of these papers to show that this method of treating the early stage of yellow-fever has been long and universally adopted; and that the opinions and practice of Drs. Jackson, Rush, Moseley, Rutherford, and Lempriere, sufficiently controvert the assumed originality of a paper on this subject by Mr. Parson, reviewed in our preceding volume. The paper No. VIII. is published with the similar view of showing that the practice suggested by Mr. Parson must have been generally known, especially as Dr. Dickson, physician to the fleet, had, in 1810, dispersed a circular on this subject to the surgeons on the Leeward Island station.

### IX. *On the Brain Fever produced by Intoxication.* *By* John Armstrong, M.D.

The disease here called *Brain Fever* appears very distinctly to be that which Dr. Sutton describes under the appellation *Delirium Tremens* (vide the Report at the beginning of this Number). Dr. Armstrong's description of the disease, and his treatment, justifies this conclusion.

"This disease," says Dr. Armstrong, "which I shall continue to designate brain-fever, is preceded by restlessness, defective recollection, paleness of the face, and slight tremors of the limbs; by anxiety, and irregularity of thought. At first the patient's slumbers are short, and interrupted by frightful dreams; but he soon becomes watchful, and passes days and nights without sleep; he dislikes to be alone, and if his friends leave him in private, he is clamorous till they return, or goes about the house in search of them. His appetite is considerably diminished, and he frequently loaths the very sight of animal food. He is more especially sick at the stomach towards the morning; he often vomits his breakfast; and the slightest exercise, or agitation of mind, produces perspiration. As the complaint advances, the skin becomes hot and dry, the tongue parched, and the pulse weak and rapid. The surface of the body, however, soon grows cooler, and is covered with sweat, and the tongue puts on a cleaner appearance; but the irregularity of mind increases; the patient imagines that his friends are all conspiring against him, or that they have suffered some great misfortune, in which he is himself deeply implicated;—at other times he supposes that his chamber is haunted by spectres, and furiously calls for assistance to drive them away; or supposes that he is in a prison, and that his friends have all deserted him; sometimes, however, he is in high spirits, laughing and talking by turns incessantly. Occasionally, too, he converses with the medical attendant about his ordinary business, with apparent precision; tells him that he has been continually engaged, and walked or rode to several places in the neighbourhood, since he last saw him, when, in reality, he had never left his own room:—at the next visit he mistakes the physician for some other person, and loads him with abuse. If any one happen to contradict him, he most pertinaciously adheres to his opinion, and becomes highly indignant. If he be soothingly dealt with, he will sometimes answer questions readily and distinctly; but if many interrogations be put to him in succession, he grows confused, and relapses into delirium.

"The symptoms already described continue more or less urgent for four, five, or six, and seldom longer than ten days. If the patient falls into a sound and tranquil sleep, he generally wakens refreshed and collected, and from that time recovers rapidly: but short disturbed slumbers, accompanied with subsultus tendinum, from which the patient starts with affright, and then falls into a low muttering delirium, are amongst the most dangerous indications. I have seen one case accompanied by convulsions from the very beginning of the disease; but they were speedily subdued by a large dose of æther, and the patient recovered very well.

"This

"This kind of brain-fever is distinguished from typhus by not being contagious; by having no petechiæ, or cadaverous smell; by the delirium attacking the patient more suddenly, and continuing with more impetuosity; by the heat of the surface of the body not remaining permanently above the natural standard; and by there being less prostration of the muscular powers in the commencement of the complaint. It is known from inflammation of the brain, by the more moderate degree of fever; by the absence of turgescence and redness of the eyes, and impatience of light; by the paleness of the face and weakness of the pulse."

The number of cases treated by Dr. Pearson of Newcastle, who published a small tract on this subject in 1801, and by Dr. Armstrong, justify, perhaps, the positive conclusion, "that this disease *invariably arises from intoxication*." The treatment which is stated by Dr. Armstrong to be generally successful, is precisely that laid down by Dr. Sutton.

"About 40 or 50 drops of laudanum should be administered on the first attack of the disease, and repeated in doses of 25 drops every five or six hours, till rest be procured. An ingenious friend of mine, who has seen much of this disease, always combines small doses of æther with the laudanum, which he has found an efficacious method of treatment. I have myself witnessed its good effects in three severe cases, and have reason to think that it is an improvement upon the practice here recommended. The common drink of the patient may be barley-water, agreeably acidulated with lemon-juice. From a pint to a bottle of Madeira wine may be allowed in twenty-four hours, regulating the quantity, however, by the state of his constitution and previous habits. Fresh ale or porter may also be moderately allowed him, and, in addition, the system must be supported by strong beef-tea and nutritious soups. Madeira is preferable to any other wine, because it rests better upon the stomach, and, in general, restores the tone of that organ in such cases, better than any other stimulus. Coercion must never be used; on the contrary, the feelings of the patient must be soothed by the kindest attentions, and he must be permitted to walk about the house, or even into the open air for a short time, if he desire it, provided the weather be sufficiently temperate. Venesection is almost invariably inadmissible, and, perhaps, highly dangerous in every case of the kind; at least I have known some instances in which it was attended with fatal consequences. Blisters never do any good, but generally harm, from the irritation which they excite. Drastic purgatives, and even mild aperients, in general, must be avoided in the commencement of the attack; but when the patient has once obtained refreshing sleep, the latter may be administered with safety, and even advantage, but the former must never be given in this disease."

### X. *On the Nature of the Disease affecting Persons employed in Silvering Mirrors. By* Edward Percival, M.D.

In a Report of the Carey-street Dispensary, in July 1812, Dr. Bateman, the reporter, states the effects produced by the the poison of mercury on the constitution, of persons employed in silvering mirrors. Dr. Percival, comparing the phenomena of these cases with the symptoms which arise invariably from the absorption of lead, pronounces that the disease described by Dr. Bateman did not arise from the poison of mercury, but from the poison of lead.

This becomes a question of high importance to the artisans engaged in silvering mirrors, for their health, sooner or later, we believe, always suffers in that employment. If, as stated by Dr. Percival, the amalgam employed is always adulterated with lead, and if that lead, not being essential to the process, be the source of the disease, the hazard of the workmen may be entirely avoided by using a purer material. The reply of Dr. Bateman will afford us the occasion to resume this subject.

XI. *Reply to Mr. Field's Justification of the Apothecaries Company, in regard to the late London Pharmacopœia. By* Richard Phillips.

We have but little inclination to enter on the ground of these criminations and recriminations. Whether the Apothecaries Company has sometimes or frequently sold imperfect preparations, or whether Mr. Phillips is always correct, and never falls into the carelessness and mistakes he charges upon that Company, we have not the means of determining.

XII. *Observations on the Case of Ann Moore. By* A. Henderson, M.D.

These observations having appeared in our Journal, enlarged and corrected by the author, we shall dismiss them with the remark, that they have been the means of bringing out a complete detection of the imposition practised by Ann Moore.

